# Does dynamic stability govern propulsive force generation in human walking?

**DOI:** 10.1098/rsos.171673

**Published:** 2017-11-29

**Authors:** Michael G. Browne, Jason R. Franz

**Affiliations:** Joint Department of Biomedical Engineering, University of North Carolina at Chapel Hill and North Carolina State University, Chapel Hill, NC, USA

**Keywords:** variability, push-off, balance, walking speed, biofeedback, ageing

## Abstract

Before succumbing to slower speeds, older adults may walk with a diminished push-off to prioritize stability over mobility. However, direct evidence for trade-offs between push-off intensity and balance control in human walking, independent of changes in speed, has remained elusive. As a critical first step, we conducted two experiments to investigate: (i) the independent effects of walking speed and propulsive force (*F*_P_) generation on dynamic stability in young adults, and (ii) the extent to which young adults prioritize dynamic stability in selecting their preferred combination of walking speed and *F*_P_ generation. Subjects walked on a force-measuring treadmill across a range of speeds as well as at constant speeds while modulating their *F*_P_ according to a visual biofeedback paradigm based on real-time force measurements. In contrast to improvements when walking slower, walking with a diminished push-off worsened dynamic stability by up to 32%. Rather, we find that young adults adopt an *F*_P_ at their preferred walking speed that maximizes dynamic stability. One implication of these findings is that the onset of a diminished push-off in old age may independently contribute to poorer balance control and precipitate slower walking speeds.

## 1. Introduction

Older adults are at a high risk of falls, and most of these falls occur during locomotor activities such as walking [1–4]. Older adults may thus opt to walk slower to improve their resilience to unexpected balance challenges and mitigate their risk of falls. Indeed, preferred walking speed decreases on average by 16% per decade after age 60 [5,6]. However, prior to eliciting slower preferred speeds, advanced age is associated with a precipitous reduction in propulsive forces (i.e. the anterior component of the ground reaction force vector, *F*_P_) exerted during the push-off phase of walking [7]. This biomechanical change is most often ascribed to sarcopenia and leg muscle weakness (though see Franz [8]), and may itself precipitate slower speeds; indeed, humans regulate walking speed by modulating propulsive forces during push-off [8,9]. As an alternative, or perhaps complementary explanation, Winter *et al*. [10] originally proposed that many of the hallmark biomechanical features of elderly gait, including reductions in propulsive force generation during push-off, reflect the adoption of a safer, more stable pattern of movement [10]. Accordingly, older adults may walk with smaller propulsive forces, even prior to walking slower, to prioritize stability over mobility. However, direct evidence for trade-offs between propulsive force generation and walking balance control, independent of changes in walking speed, has remained elusive.

There is no consensus in the literature as to which metrics best describe the integrity of walking balance control. However, local dynamic stability quantified via maximum divergence exponents can provide unique insight, distinguishes between older and young adults and correlates with age-related falls risk [11–13]. Here, we use balance as a general term describing the resilience to falling and operationally define dynamic stability as a specific metric of balance. The cumulative insights from Dingwell & Marin [12] and Kang & Dingwell [13] suggest that by walking slower, both young and older adults can improve their local dynamic stability [12,13]. Moreover, their subjects did so while successfully accommodating an increase in kinematic variability—another frequently employed metric of walking balance control that is known to increase with slower speeds [14]. These results are not unequivocal, however. Indeed, some studies suggest that speed has no effect on dynamic stability [15] or that results may vary depending on calculation method [16]. Though, when faced with cognitive or physical balance challenges, older adults do prefer to reduce their walking speed [17]. Thus, despite some conflicting reports, we posit that it is reasonably well conceived that older adults may ultimately walk slower to mitigate their risk of falls. Ultimately, there may be a complex interdependence between dynamic stability and simultaneous age-associated biomechanical changes also thought to precipitate slower speeds.

Also preceding and perhaps contributing to slower walking speeds in old age, the single most common biomechanical change in elderly gait is up to 20% reduction in propulsive forces exerted during push-off compared to young adults walking at the same speed [7]. Underlying these smaller propulsive forces is an 11–35% reduction in mechanical power generated by the propulsive plantarflexor (i.e. ankle extensor) muscles [18,19]. Appropriate ankle power generation is critical in walking, at once contributing to leg swing and centre of mass (CoM) acceleration [20]. Moreover, we consider *F*_P_ and ankle power generation inextricably linked; peak ankle power is a significant contributor to peak *F*_P_ [21] and walking with smaller *F*_P_ systematically decreases peak ankle power [22]. Although commonly implicated, sarcopenia and muscle weakness alone cannot explain these biomechanical changes associated with ageing; after accounting for declines in muscle-force generating capacity, between 48 and 75% of the variance in ankle power and thus propulsive force generation is left unexplained [23]. In addition, the appropriate modulation of ankle power has also been shown to be important for stabilizing computational models of walking, thereby alluding to a potentially important relation between push-off intensity and balance control [24].

Do older adults change their gait biomechanics, prior to walking slower, to mitigate the risk of falls? In their seminal paper, Winter *et al*. [10] alluded to the presence of trade-offs between propulsive force generation during push-off and walking balance control [10]. Specifically, those authors suggested that ‘a normal push-off… is a thrust from the ankle, which acts upward and forward, and is destabilizing. The elderly… appear to have recognized this fact and are reducing that potential for instability.’ The presence of this trade-off in human locomotion has yet to be experimentally validated, but could manifest in two ways. First, and most consistent with the context provided by Winter *et al*. [10], walking at a given speed with smaller propulsive forces could improve dynamic stability. Second, the freely selected magnitude of propulsive force generation while walking at a preferred speed could maximize dynamic stability. As a critical first step, we sought to investigate the presence of this trade-off in young adults, and thus, the role of dynamic stability in governing propulsive force generation during walking in the absence of age-associated gait changes.

Therefore, in two experiments, the purposes of this study were to investigate: (i) the independent effects of walking speed and propulsive force generation on dynamic stability in young adults, and (ii) the extent to which young adults prioritize dynamic stability in selecting their preferred combination of walking speed and propulsive force generation. We tested two independent hypotheses that were, at least on the surface, mutually exclusive. First, we hypothesized that walking slower or with smaller propulsive forces would improve dynamic stability. Second, we hypothesized that young adults prefer a combination of walking speed and propulsive force generation that maximizes dynamic stability. We also analysed step kinematics and metrics of gait variability in each test of our hypotheses, given their complement to measures of dynamic stability within the broader context of walking balance control.

## 2. Methods

### 2.1. Subjects

We present data from healthy young adult subjects that participated in two different but complementary experiments which we refer to as *experiment 1* and *experiment 2*, both outlined in detailed below; 12 subjects participated in experiment 1 (mean ± s.d., age: 26.2 ± 3.1 years, height: 1.75 ± 0.09 m, mass: 71.6 ± 8.8 kg, six males/six females) and 10 subjects participated in experiment 2 (age: 24.8 ± 5.4 years, height: 1.78 ± 0.08 m, mass: 73.2 ± 7.6 kg, five males, five females). All subjects provided written, informed consent according to the University of North Carolina Institutional Review Board.

### 2.2. Visual biofeedback paradigm

**Figure 1.**
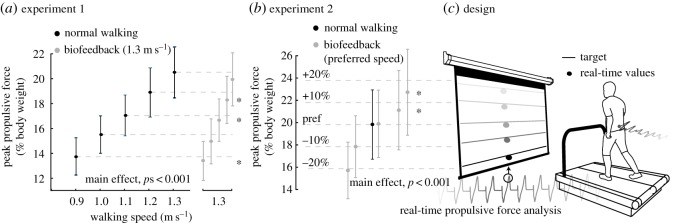
Group mean (standard deviation) peak propulsive force values (*a*) when walking across a range of speeds and at 1.3 m s^−1^ with biofeedback of propulsive force targets extracted from slower speeds and (*b*) walking at preferred speed with propulsive forces ±10% and ±20% larger than preferred. (*c*) Experimental design using visual biofeedback of real-time propulsive force values calculated from a dual-belt, force-measuring treadmill to decouple the effects of walking speed and propulsive force generation on metrics of dynamic balance control. Asterisks represent significant (*p* < 0.05) difference from prescribed values.

Experiment 1 and experiment 2 both used a novel visual biofeedback paradigm based on real-time force measurements from a dual-belt force measuring treadmill (Bertec, Corp., Columbus, OH, USA) (figure 1*c*). Specifically, for trials involving this biofeedback paradigm, a custom Matlab (Mathworks, Natick, MA, USA) script continuously computed the average bilateral peak horizontal (i.e. propulsive, *F*_P_) force during push-off from each set of four consecutive steps and projected a visual representation of those values as dots in real time to a screen positioned in front of the treadmill (figure 1*c*). To ensure the visual feedback was as intuitive as possible, we described to each subject the timing of push-off and explained that *F*_P_ represented the force on the ground acting to accelerate their body forward with each step. Subjects were then encouraged to match their instantaneous *F*_P_ to target values displayed as horizontal lines and prescribed according to the experimental protocols outlined below. For all trials involving visual biofeedback, we normalized the scaling of each subject's feedback data on the projected display so all target values were evenly distributed over the ordinate range.

### 2.3. Experiment 1 protocol

We reanalysed data available from a previously published protocol outlined in detail elsewhere [22]. To summarize briefly, subjects first walked normally on the force-sensing treadmill for 1 min each at five different speeds (0.9, 1.0, 1.1, 1.2 and 1.3 m s^−1^), in randomized order. We extracted each subject's bilateral peak *F*_P_ from each walking speed, which were subsequently used as target values for visual biofeedback trials. Subjects then completed a 5 min exploratory walking session in which they became familiar with the biofeedback environment. Finally, subjects walked at 1.3 m s^−1^ for 1 min each while matching their instantaneous *F*_P_ to the mean values extracted from all five walking speeds.

### 2.4. Experiment 2 protocol

A photo cell timing system first assessed subjects’ preferred overground walking speed from the average of three times taken to traverse the middle 2 m of a 10 m walkway when asked to walk at a normal, comfortable speed (1.27 ± 0.14 m s^−1^) (Brower Timing, Draper, UT, USA). Subjects then walked at their preferred overground speed normally and while matching a randomized series of biofeedback targets on the force-sensing treadmill. Specifically, subjects walked while matching their instantaneous *F*_P_ to values representing the mean value and ±10% and ±20% of the mean value extracted from the normal walking trial.

### 2.5. Data collection and analysis

A dual-belt, force-sensing treadmill (Bertec, Inc.) operating at 1000 Hz recorded the right and left leg ground reaction forces, used in the protocol as described in the prior sections. In synchrony, a 14-camera motion capture system (Motion Analysis, Corp., Santa Rosa, CA, USA) operating at 100 Hz recorded the three-dimensional trajectories of markers placed on subjects' pelvis and right and left legs. Our analyses focused on a reduced set of these markers, the sacrum and right and left heel trajectories, which we low-pass filtered using a fourth-order Butterworth filter and a cut-off frequency of 12 Hz. Using previously published methods, we extracted the instants of right and left heel-strikes from the peak anterior heel positions relative to the sacral marker [25], which we then used to calculate time series of step widths and lengths as follows. We calculated step width using the average mediolateral distance between heel marker positions during midstance prior to heel rise (i.e. 12–25% of the gait cycle) across successive steps. Accordingly, we derived step width variability as the standard deviation of the step width time series. We calculated step length using the relative anterior–posterior positions of successive heel markers at 20% of the gait cycle plus the treadmill belt translation over each step. Step length variability was the corresponding standard deviation of those step length time series.

We used the filtered, three-dimensional sacrum trajectory components as a surrogate for subjects’ CoM, from which we calculated two kinematically derived metrics of walking balance control—variability and local dynamic stability. Time series of sacrum position can exhibit non-stationarity arising from subjects' average position changing over the course of a walking trial. This non-stationarity can influence metrics of movement variability but may also contain relevant information about walking balance that we chose not to disregard. Thus, we calculated CoM variability in the anterior–posterior, mediolateral and vertical directions using the standard deviation of both: (i) the sacrum position time series, and (ii) the sacrum velocity time series, the latter being less affected by changes in average CoM position.

Finally, we used time series of sacrum position and velocity components to calculate maximum divergence (Lyapunov) exponents. These exponents, which quantify the sensitivity of CoM motion to small, naturally occurring perturbations arising from internal (e.g. neuromuscular noise) and external (e.g. biofeedback) factors, served as our metric of local dynamic stability. To do this, we first reconstructed state spaces, *S*(*t*), from the original data in the anterior–posterior (*x*), mediolateral (*y*) and vertical (*z*) directions and their time-delayed copies according to the following equations

2.1 $$\begin{array}{rl} & q_{\mathrm{anterior}\text{–}\mathrm{posterior}}(t)=[x,\dot{x\;}\;\;], \end{array}$$

2.2 $$\begin{array}{rl} & q_{\mathrm{mediolateral}}(t)=[y,\dot{y}], \end{array}$$

2.3 $$\begin{array}{rl} & q_{\mathrm{vertical}}(t)=[z,\dot{z}], \end{array}$$

2.4 $$\begin{array}{rl} & q_{3D}(t)=[x,y,z,\dot{x\;}\;\;,\dot{y},\dot{z}] \end{array}$$

2.5 $$\begin{array}{rl} \text{and}\; & \text{S}(t)=[q(t),q(t+\tau ),q(t+2\tau ),q(t+3\tau ),q(t+4\tau ),q(t+5\tau )], \end{array}$$

where ‘3D’ in equation (2.4) refers to a composite metric assembled from the anterior–posterior, mediolateral and vertical components. For each of these four state space reconstructions for each subject, we calculated the average maximum exponential rates of divergence of pairs of initially neighbouring trajectories using procedures outlined in detail previously [13,26,27]. Consistent with our prior work [28], we used one-quarter of subjects' average stride time as the embedding delay, *τ*, for all conditions [29], and determined the corresponding embedding dimension (*d*_E_ = 5) using a 10% criterion in a false nearest neighbours analysis [30]. After time normalizing the divergence curves to account for differences in stride period, we calculated each subject's short-term (*λ*_s_, 0–1 stride) divergence exponents, where larger positive values imply larger local *instability*. For the purposes of this study, we opted not to also analyse long-term divergence exponents (e.g. 1–10 strides), as we and others have found these to be largely insensitive to between-group or between-condition effects during treadmill walking [13,28]. Indeed, walking on a treadmill at a constant speed requires that subjects' movements remain loosely bounded over the course of many strides. Note that throughout the Results and Discussion, better dynamic stability refers to smaller values of *λ*_s_.

### 2.6. Statistical analysis

The paired-sample *t*-tests performed on subjects’ average peak propulsive force first determined the success of subjects reaching *F*_P_ biofeedback targets. We then performed separate statistical analyses on the following outcome measures collected in experiments 1 and 2: step length and width, step length and width variabilities, and the three-dimensional variabilities and short-term local divergence exponents (i.e. dynamic stability) derived from sacrum marker trajectories. For experiment 1, a two-way repeated-measures analysis of variance (ANOVA) tested for significant main effects of and interactions between condition (i.e. normal walking versus biofeedback) and speed-matched targets (i.e. 0.9–1.3 m s^−1^). When a significant interaction was found, Fisher's LSD *post hoc* comparisons elucidated the speed-matched targets at which differences emerged. For experiment 2, paired-samples *t*-tests tested for effects of biofeedback alone on each outcome measure by comparing normal walking to walking with the biofeedback target representing *F*_P_ during normal walking. Then, a one-way repeated-measures ANOVA tested for a significant main effect of *F*_P_ (i.e. 0, ±10%, ±20%) on each outcome measure. When a significant main effect was found, planned Fisher's LSD *post hoc* comparisons were focused to elucidate significant differences from normal walking (with and without biofeedback).

## 3. Results

Subjects successfully and systematically modulated their *F*_P_ in accordance with all prescribed biofeedback targets presented in both experiments 1 and 2 (figure 1*a*,*b*). In addition, biofeedback itself (i.e. normal walking versus walking with targets representing normal walking) had only small discernible effects on our outcome measures; *post hoc* comparisons revealed that the use of biofeedback itself significantly increased only mediolateral sacrum position variability in both experiments and anterior–posterior sacrum position variability only in experiment 2 (*p* < 0.02) (figure 4).

### 3.1. Experiment 1: the independent effects of walking speed and propulsive force

**Figure 2.**
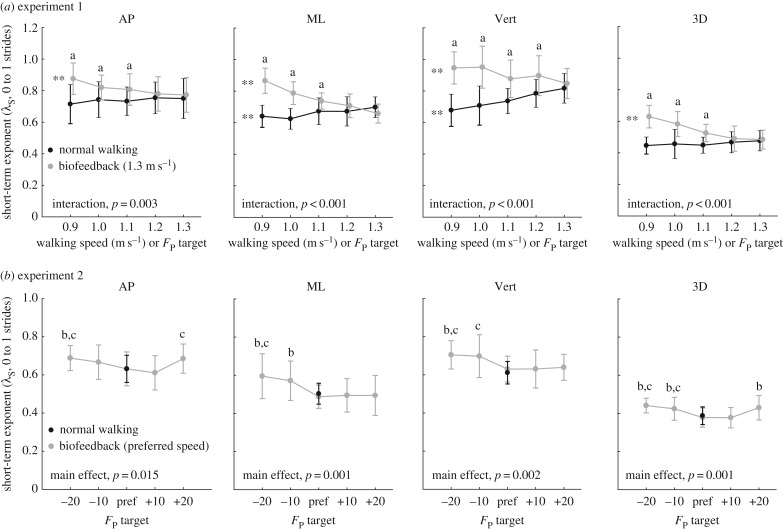
Group mean (standard deviation) short-term maximum divergence exponents (*λ*_S_) defined using anterior–posterior (AP), mediolateral (ML), vertical (Vert) and three-dimensional (3D) sacrum kinematics for (*a*) systematic changes in walking speed and propulsive force targets extracted from slower speeds, and (*b*) walking at preferred speed with propulsive forces smaller or larger than preferred. Double asterisks represent significant (*p* < 0.05) main effects of speed or propulsive force. ‘a’ Indicates significant (*p* < 0.05) pairwise difference at matched propulsive forces, ‘b’ indicates significantly (*p* < 0.05) different from normal walking with biofeedback, and ‘c’ indicates significantly (*p* < 0.05) different from normal walking without biofeedback.

**Figure 3.**
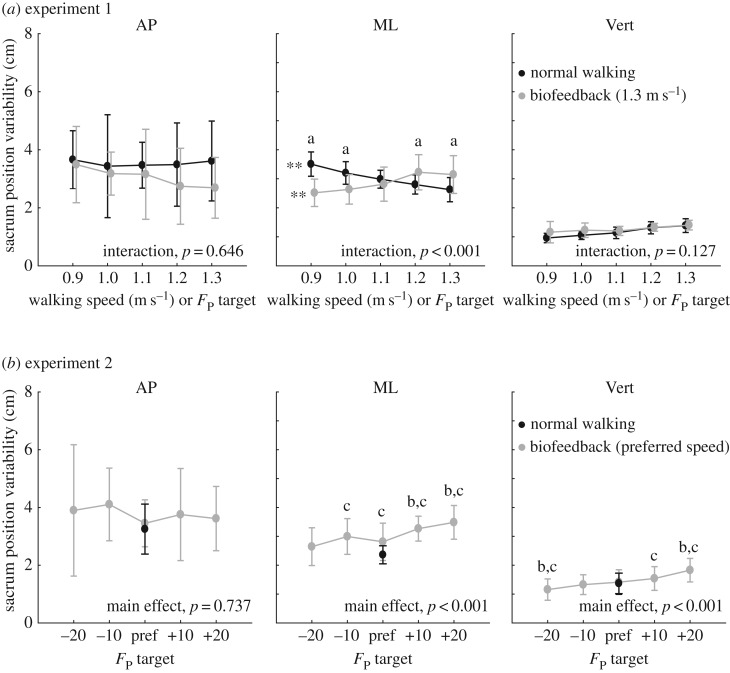
Group mean (standard deviation) anterior–posterior (AP), mediolateral (ML) and vertical (Vert) sacrum position variability for (*a*) systematic changes in walking speed and propulsive force targets extracted from slower speeds, and (*b*) walking at preferred speed with propulsive forces smaller or larger than preferred. Double asterisks represent significant (*p* < 0.05) main effects of speed or propulsive force. ‘a’ Significant (*p* < 0.05) pairwise difference at matched propulsive forces, ‘b’ significantly (*p* < 0.05) different from normal walking with biofeedback and ‘c’ significantly (*p* < 0.05) different from normal walking without biofeedback.

**Figure 4.**
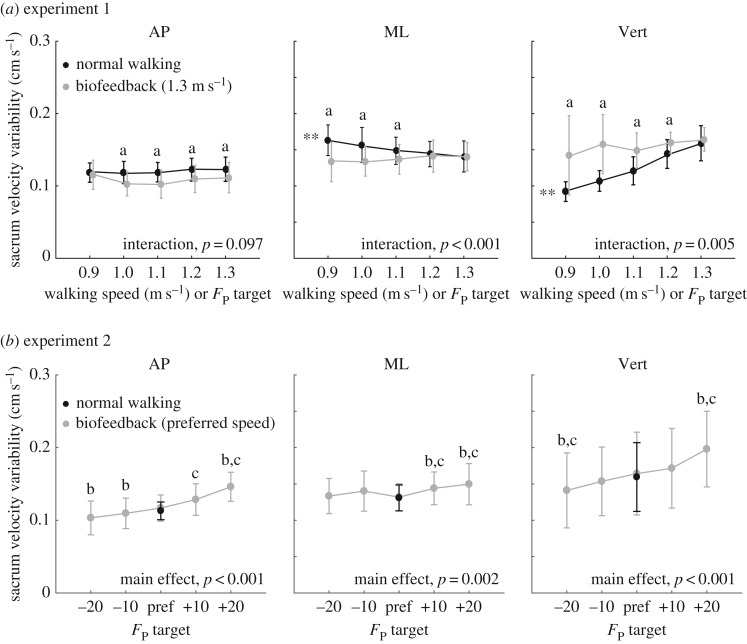
Group mean (standard deviation) anterior–posterior (AP), mediolateral (ML) and vertical (Vert) sacrum velocity variability for (*a*) systematic changes in walking speed and propulsive force targets extracted from slower speeds, and (*b*) walking at preferred speed with propulsive forces smaller or larger than preferred. Double asterisks represent significant (*p* < 0.05) main effects of speed or propulsive force. ‘a’ Significant (*p* < 0.05) pairwise difference at matched propulsive forces, ‘b’ significantly (*p* < 0.05) different from normal walking with biofeedback and ‘c’ significantly (*p* < 0.05) different from normal walking without biofeedback.

Walking slower and walking with smaller *F*_P_ elicited very different and direction-dependent effects on short-term local divergence exponents and gait variability. Walking slower decreased local divergence exponents by an average of up to 8% in the mediolateral direction (*p* = 0.005) and 21% in the vertical direction (*p* < 0.001) across the range of speeds tested (figure 2*a*). This improved stability was accompanied by significant increases in mediolateral sacrum variability (position: *p* < 0.001, velocity: *p* < 0.001) (figures 3*a* and 4*a*) yet significant decreases in vertical sacrum variability (velocity: *p* < 0.001) (figure 4*a*). Finally, walking slower had no significant effect on step width or step width variability, but did elicit progressively shorter and more variable step lengths (step length: *p* < 0.001; step length variability: *p* < 0.001) (table 1). In contrast with walking slower, and despite exerting identical *F*_P_ during push-off, walking at 1.3 m s^−1^ while independently reducing *F*_P_ using biofeedback elicited significant deficits in dynamic stability. Specifically, walking with smaller *F*_P_ increased short-term local divergence exponents by an average of up to 13, 32 and 12%, in the anterior–posterior, mediolateral and vertical directions, respectively, and up to 30% for the three-dimensional metric (figure 2*a*) (pairwise, *p*s < 0.023). Moreover, significant interactions revealed that these effects differed significantly from those due to walking slower (*p*s < 0.003). These stability deficits were accompanied by a significant decrease in mediolateral sacrum variability (position: *p* < 0.001), an effect that also differed significantly from walking slower (interaction, *p* < 0.001) (figure 3*a*). Finally, similar to walking slower, walking with reduced *F*_P_ also elicited progressively shorter and more variable step lengths (step length: *p* < 0.001; step length variability: *p* = 0.006) (table 1).

**Table 1.** Mean ± s.d. step kinematics (centimetres). SL, step length; SW, step width; SLV, step length variability; SWV, step width variability.

*experiment 1*						
normal walking (speed, m s^−1^)						
	0.9	1.0	1.1	1.2	1.3	main effect
SL	57.38 ± 3.01	−60.32 ± 3.19	63.29 ± 3.49	66.86 ± 3.12	69.60 ± 3.31	*p < 0.001*
SW	16.07 ± 2.68	15.71 ± 3.05	15.61 ± 2.84	15.16 ± 2.39	15.22 ± 2.63	*p = 0.036*
SLV	2.44 ± 0.56	2.23 ± 0.62	1.98 ± 0.64	2.05 ± 0.72	1.98 ± 0.78	*p = 0.002*
SWV	1.72 ± 0.44	1.79 ± 0.32	1.75 ± 0.38	1.84 ± 0.38	1.84 ± 0.35	*p* = 0.373
biofeedback at 1.3 m s^−1^ (speed-matched propulsive force target)						
	0.9	1.0	1.1	1.2	1.3	
SL	56.65 ± 3.75	59.11 ± 3.41	61.79 ± 3.17	65.97 ± 3.05	67.74 ± 3.03	*p < 0.001*
SW	15.13 ± 1.65	15.34 ± 2.08	15.08 ± 1.71	15.41 ± 2.11	15.41 ± 1.86	*p* = 0.846
SLV	2.97 ± 0.74	2.55 ± 0.64	2.48 ± 0.73	2.18 ± 0.73	1.99 ± 1.02	*p = 0.002*
SWV	1.82 ± 0.44	1.88 ± 0.32	1.71 ± 0.30	1.78 ± 0.36	1.77 ± 0.27	*p* = 0.394
*experiment 2*						
propulsive force target						
	−20%	−10%	normal	+10%	+20%	
SL	62.37 ± 6.51^a^^,^^b^	65.44 ± 6.9^a^	67.8 ± 6.10	69.93 ± 6.41	71.9 ± 6.93	*p < 0.001*
SW	17.05 ± 2.94	17.12 ± 2.65^a^^,^^b^	15.66 ± 2.99	17.82 ± 2.69^a^^,^^b^	18.78 ± 2.79^a^^,^^b^	*p < 0.001*
SLV	2.55 ± 0.48^a^^,^^b^	2.53 ± 0.61^a^^,^^b^	2.02 ± 0.53	2.58 ± 0.72	3.47 ± 0.93^a^^,^^b^	*p < 0.001*
SWV	1.59 ± 0.29	1.63 ± 0.27	1.82 ± 0.54	1.87 ± 0.33	1.90 ± 0.40	*p* = 0.103

^a^Significantly (*p* < 0.05) different from normal walking with biofeedback.

^b^Significantly (*p* < 0.05) different from normal walking without biofeedback.

### 3.2. Experiment 2: the preferred combination of walking speed and propulsive force

Deviating from the *F*_P_ subjects exerted when walking normally at their preferred speed negatively affected metrics of dynamic stability (figure 2*b*). Consistent with experiment 1 results, walking with smaller than preferred *F*_P_ increased short-term local divergence exponents in all directions. Here, walking with 20% larger than preferred *F*_P_ also increased short-term local divergence exponents, by 8% in the anterior–posterior direction (*p* = 0.017) and by 14% for the three-dimensional metric (*p* = 0.041). By contrast, all components of sacrum variability decreased monotonically with smaller *F*_P_ across the range of biofeedback targets presented (figures 3*b* and 4*b*). Finally, like the short-term local divergence exponents, we identified a local minimum also in step width and step length variability at subjects’ preferred combination of walking speed and *F*_P_ (table 1). For example, walking with 20% larger (smaller) than preferred *F*_P_ increased step width by 20% (9%) and step length variability by 71% (26%) (pairwise, *p*s < 0.01).

## 4. Discussion

Humans modulate their walking speed using propulsive forces generated during push-off. Accordingly, slower preferred speeds in old age are accompanied and preceded by smaller peak *F*_P_ during push-off compared to young adults, and both may represent an effort to mitigate instability and risk of falls. Indeed, Winter *et al.* [10] suggested in their seminal paper that older adults may reduce their push-off intensity prior to walking slower to alleviate the potential for instability [10]. However, although highly intuitive, direct evidence for trade-offs between *F*_P_ generation and walking balance control, independent of changes in walking speed, has remained elusive. Our findings provide the first empirical evidence that walking slower and walking with smaller *F*_P_ elicit very different effects on local dynamic stability, at least in young adults. Although some prior studies differ in their conclusions [15], consistent with works of Dingwell & Marin [12] and that of Kang & Dingwell [13], we found here that walking slower improved dynamic stability despite increasing movement variability [12–14]. However, contrary to our first hypothesis, walking with smaller *F*_P_ reduced dynamic stability compared to walking normally. Rather, more consistent with our second hypothesis, our findings suggest that young adults adopt a push-off intensity at their preferred walking speed that maximizes dynamic stability. Cumulatively, and as we elaborate in more detail below, our findings allude to unfavourable consequences of reduced propulsive force generation on dynamic stability that may ultimately precipitate walking slower.

### 4.1. Independent effects of walking speed and *F*_P_ on dynamic balance control

Motivated most directly by the contextual premise of Winter *et al*. [10], we first hypothesized that walking slower or with smaller *F*_P_ would improve dynamic stability. More precisely, in this study, we found that walking slower, but not with smaller *F*_P_, improved dynamic stability. Walking slower decreased short-term divergence exponents in the mediolateral and vertical directions despite increasing movement variability, largely consistent with the findings of Dingwell & Marin [12]. Moreover, the direction-dependent effects here are not entirely surprising. Unlike lateral balance, which relies heavily on active control and is disproportionately susceptible to perturbations, walking affords some passive stability and resistance to perturbations in the direction of movement [31–35]. Indeed, in contrast with our hypothesis and with walking slower, walking with smaller *F*_P_ reduced dynamic stability by up to 32%, with the largest effects in the mediolateral direction.

Metrics of kinematic variability, quantifying the magnitude of step-to-step adjustments in walking, provide an important complement to measures of dynamic stability within the broader context of walking balance control. Too little or too much variability has been implicated as a marker of walking balance deficits and, although findings differ by study, both correlate with a history of falls [36]. Consistent with some previous work [12,14], walking slower increased movement variability—here, that of step length and mediolateral sacrum kinematics. The most likely interpretation is that walking slower brings a reduced requirement for strict step-to-step kinematic control to maintain dynamic stability. Walking with reduced *F*_P_ had comparable effects on two variability outcome measures. The first, mediolateral sacrum position variability, also increased with the use of biofeedback alone (i.e. biofeedback of normal *F*_P_) and may reflect the subject's global position on the treadmill more than step-to-step adjustments associated with balance control. The second, step length variability, may be related to larger step-to-step adjustments in propulsive force as subjects attempted to regulate their values to match the targets from slower speeds. Indeed, propulsive forces generated during push-off may govern walking speed via changes in step length. However, biofeedback alone had no appreciable effect on step length variability.

### 4.2. The stability maximization hypothesis

The second way in which trade-offs between propulsive force generation during push-off and walking balance control could manifest, informing our second hypothesis and the design of experiment 2, was that young adults could prefer a combination of walking speed and *F*_P_ generation that maximizes dynamic stability. Our results are consistent with this hypothesis; we found evidence of a local minimum in local divergence exponents at the *F*_P_ subjects normally exerted when walking at their preferred speed. Walking with smaller than preferred *F*_P_ in experiment 2 yielded reductions in dynamic stability that were highly consistent with those in experiment 1. Experiment 2 added that walking with larger than preferred *F*_P_, at least for increases of 20%, also reduced dynamic stability, specifically that in the anterior–posterior direction. Thus, we interpret our results to suggest that young adults elect an *F*_P_ at their preferred speed that maximizes their local dynamic stability. This is not an entirely novel proposition; step-to-step ankle power control of robotic prostheses can improve walking balance performance [24,37]. Here, recall that push-off contributes to both leg swing and CoM acceleration [20], thus manipulating push-off intensity could have negative effects on stability by way of step-to-step disruptions to leg swing and/or CoM acceleration. Interestingly, dynamic stability does seem to be somewhat less susceptible to increasing push-off intensity; increasing *F*_P_ by 10% had no effect on dynamic stability compared to normal walking. One possible explanation is that increases in push-off intensity were simply dissipated by the swing leg with negligible impacts on CoM acceleration. We also discovered that changes in dynamic stability across conditions were direction-dependent; only that derived from anterior–posterior sacrum trajectories was susceptible to the 20% increase in *F*_P_. This may suggest a resilience to increased push-off intensity in the mediolateral and vertical directions.

Young adults have a well-documented affinity for optimizing in their locomotor patterns [38–40]. For example, young adults adopt step frequencies [41] and step widths [33] that minimize metabolic costs. These studies have led to an energy minimization hypothesis governing human locomotion—that healthy individuals adopt locomotor patterns in walking that minimize metabolic energy consumption. Similarly, based on our current findings, we posit that stability maximization may also play a role in governing locomotor patterns, at least in young adults' selection of their preferred push-off intensity. Ultimately, the locomotor pattern which optimizes push-off intensity and balance and that which optimizes metabolic energy expenditure may not be mutually exclusive. Indeed, the naturally emergent timing and magnitude of a propulsive push-off from the ankle plantarflexor muscles is known to contribute to economical walking [42]. Although we did not measure rates of oxygen consumption, doing so would be a valuable contribution to similar studies in the future.

### 4.3. Decoupling changes in kinematic variability versus dynamic stability

Increasing propulsive forces beyond their preferred magnitude in experiment 2 elicited fundamentally different changes in the relation between dynamic stability and movement variability compared to the disparate effects in these outcome measures reported for walking faster. When walking faster than preferred, humans exhibit less variability, despite poorer dynamic stability [13,14]. Thus, in this case, decreased dynamic stability is associated with more tightly regulated step-to-step kinematic patterns. By contrast, when our subjects walked with larger *F*_P_ than preferred, declines in dynamic stability were accompanied by pervasive *increases* in sacrum and step length variabilities. These results have interesting implications for interpreting changes in these two commonly used metrics of walking balance control. Specifically, our results suggest that changes in dynamic stability and those in kinematic variability in human walking need not always vary in opposite directions (i.e. dynamic stability increasing and kinematic variability decreasing or vice versa). There are at least two potentially related explanations for the increased variability that accompanied walking with larger than preferred *F*_P_. First, evidence from the motor control literature has shown that executing tasks using larger muscle force magnitudes yields larger force fluctuations (i.e. increased variability) [43]. Further, Roos & Dingwell [44] used computational models to demonstrate that neuromuscular noise, a factor that increases with greater muscle activation, also increases kinematic variability [44]. Thus, at least in young adults, walking with larger *F*_P_ may increase kinematic variability via potentially interdependent changes in force production and neuromuscular noise.

### 4.4. Step length changes

**Figure 5.**
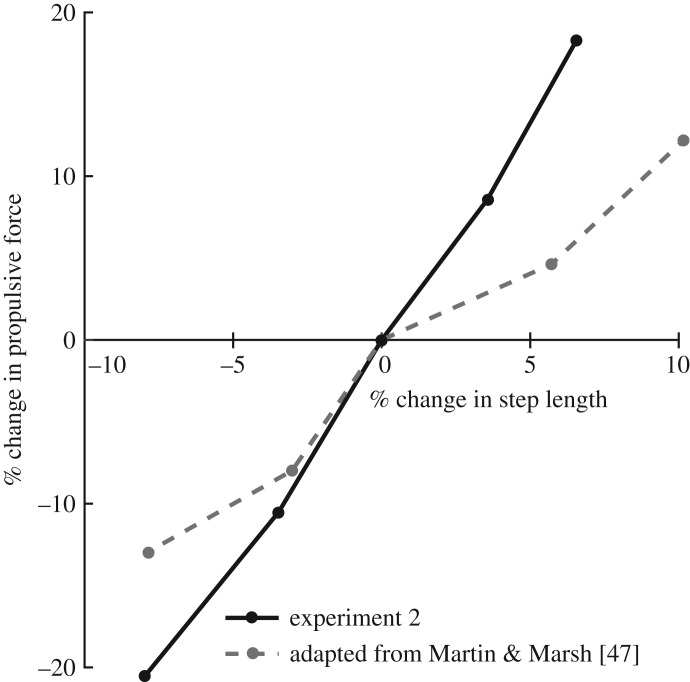
Group mean per cent change in peak propulsive force versus per cent change in step length from experiment 2, plotted against data adapted from Martin & Marsh [47]. We found that subjects increased (decreased) their step lengths by 6.6% (−8.0%) when targeting a 20% increase (decrease) in *F*_P_, compared with walking normally. Conversely, Martin & Marsh [47] found that their subjects increased (decreased) *F*_P_ by only 12.2% (−13.0%) when directly increasing (decreasing) their step lengths by 10.2% (−7.9%). These results imply that, although both change simultaneously, modifying propulsive forces is not biomechanically equivalent to modifying step lengths. Accordingly, we interpret our findings to allude to direct effects of modulating push-off intensity and not solely a result of secondary changes in step length.

Our subjects systematically increased and decreased their step lengths, on average, to increase and decrease their push-off intensity via *F*_P_ targets, respectively. Prior work has shown that, when walking at a constant speed, modulating step length via a metronome elicits changes in short-term divergence exponents in young adults, with direction-dependent changes that are relatively consistent with our findings [45]. However, we suspect that our findings reveal novel insights into walking balance control that are unique to changes in propulsive force magnitude. Indeed, modulating step length using a metronome has altogether different effects on walking biomechanics compared to directly modulating *F*_P_ [46]. For example, Martin & Marsh [47] found that increasing step length by 10% yielded a 12% increase in *F*_P_ [47]. Conversely, we found here that increasing *F*_P_ by 20% yielded a much more subtle 6% increase in step length (figure 5). In addition, independently reducing *F*_P_ at a constant walking speed elicits changes in leg joint power generation that may differ from those elicited by changes to step length alone [22,48]. Thus, although further study is required to make definitive conclusions, we interpret our findings to allude to direct effects of modulating push-off intensity and not solely the result of secondary changes in step lengths.

### 4.5. Limitations

We acknowledge several limitations of this study. First, we describe the implications of our findings primarily in the context of their relevance to age-related gait changes, but have not yet included a cohort of older adults in our study design. Second, we did not test speeds faster than preferred and instead refer to other studies for these data. Third, we conclude that a reduction in *F*_P_ during walking at a fixed preferred speed would not confer better dynamic stability. However, we cannot exclude the possibility that a diminished push-off, by way of reducing shear forces, may mitigate the risk of slipping. We do note that Winter *et al*. [10] indirectly described trade-offs between push-off intensity and walking balance control, not in the context of slips, but to explain biomechanical changes in elderly gait that occur also in controlled laboratory conditions where slips are highly unlikely. We also acknowledge that push-off intensity is often described as an upward and forward thrust from the ankle. While we admittedly only modulated forward propulsion via *F*_P_, we have shown that *F*_P_ modulation also systematically effects trailing limb CoM work and ankle power generation during push-off [22]. In addition, we interpret changes in our outcome measures elicited through the use of biofeedback to directly reflect those due to modulating *F*_P_. Indeed, subjects’ response to biofeedback alone, and thus the effects of making step-to-step adjustments to match targets, had no effect on dynamic stability. Additionally, the reader may opt to interpret our results conservatively; our *post hoc* analysis plan did not include correcting for multiple comparisons. Although we took great care in our data analysis procedures, our results may also be sensitive to methodological choices for estimating local divergence exponents which may account for some differing results between studies. For example, we implemented the Rosenstein algorithm, which functions well with smaller datasets (i.e. approx. 1 min) though loses some sensitivity for detecting differences from longer time series [49]. Lastly, there are numerous metrics for quantifying the integrity of balance control during walking. We elected to use dynamic stability and kinematic variability as our primary metrics, though Floquet multipliers [50] and margin of stability [51] may be valuable for follow-on analyses.

### 4.6. Implications for biomechanical changes in elderly gait

Older adults often walk slower than young adults. However, prior to preferring slower speeds, older adults walk with a diminished push-off—decreased *F*_P_ accompanied by reduced ankle moment and power generation. Although the mechanisms governing the onset of these biomechanical changes and how they precipitate slower preferred speeds are poorly understood and probably multi-factorial, our present findings allude to a novel explanation that warrants further study. Here, we provide empirical evidence that walking at the same speed but with a diminished push-off systematically decreases dynamic stability. Perhaps, it should not be surprising then that age-related reductions in push-off intensity are regularly accompanied by considerable reductions in dynamic stability, all before older adults choose to walk slower [9]. For example, Kang & Dingwell [14] found that older adults averaged approximately 67% worse dynamic stability than young adults, despite walking at the same preferred speed. Taken together, one interpretation of these findings is that the onset of a diminished push-off in old age may independently contribute to poorer balance control in walking. Accordingly, based on our work and that of others, these negative effects on balance control may subsequently precipitate a decrease in the preferred walking speed of older adults in their effort to restore dynamic stability. Finally, increasing a diminished push-off is a common target for interventions aimed at improving walking performance and independence, both in older adults and in people with more acute gait disability. Our work suggests that evaluating the efficacy of these interventions should include the potentially complex effects on walking balance control.

## Supplementary Material

Subject Data.xlsx

## Data accessibility

We have made all supporting data for the experiments presented in this study available in the electronic supplementary material file.
